# Deletion of RhoGDI Protects Against Hepatic Steatosis via Improved Mitochondrial Metabolism in Mice

**DOI:** 10.3390/ijms27031161

**Published:** 2026-01-23

**Authors:** Yongzhi Wang, Yuanqi Zhou, Yifan Xu, Chen Wang, Shuo Meng, Honglin Li, Huifang Tang, Jian Zhang

**Affiliations:** 1 Shanghai Frontiers Science Center of Optogenetic Techniques for Cell Metabolism, Shanghai Key Laboratory of New Drug Design, School of Pharmacy, East China University of Science & Technology, Shanghai 200237, China; 2 Key Laboratory of Multi-Omics and Artificial Intelligence of Cardiovascular Diseases, Clinical Research Center for Myocardial Injury in Hunan Province, University of South China, Hengyang 421001, China

**Keywords:** RhoGDI, MASLD, mitochondrial dysfunction, AMPK, lipid metabolism

## Abstract

The global incidence of metabolic dysfunction-associated steatotic liver disease (MASLD) is rising alongside epidemics of diabetes and obesity. Rho GDP-dissociation inhibitor (RhoGDI) is now recognized to play dual regulatory roles in disease. A deeper understanding of its mechanistic contributions in MASLD could offer critical insights for developing novel therapies against this growing health burden. Immunohistochemical staining was used to examine RhoGDI expression in liver tissues from patients with MASLD. Hepatocyte-specific deletion of Arhgdia (the gene encodes RhoGDI) was generated in mice, and they subjected to NASH diets to induce hepatic steatosis. Transcriptomic sequencing was carried out to identify altered pathways in the Arhgdia-deficient mice, followed by functional investigations of downstream signaling and mitochondrial performance. Finally, the therapeutic potential of a candidate compound was evaluated in the MASLD model. The expression level of RhoGDI was significantly upregulated, and hepatocyte-specific deletion of Arhgdia (the gene encodes RhoGDI) attenuated hepatic lipid accumulation and fibrotic progression. The RNA sequencing analysis revealed that RhoGDI deficiency suppressed the hepatic steroid hormone biosynthesis pathway. It was demonstrated that RhoGDI plays a crucial role in maintaining mitochondrial function, since hepatocyte-specific knockout of Arhgdia significantly reversed mitochondrial dysfunction in mice. Furthermore, a natural compound was found to alleviate hepatic steatosis and inflammation in MASLD mice by targeting RhoGDI. This finding demonstrates that Arhgdia deletion confers protection against the progression of MASLD by reducing hepatic lipid accumulation and enhances mitochondrial β-oxidation in hepatocytes establishing RhoGDI as a critical regulator of MASLD pathogenesis and highlighting its potential as a therapeutic target for metabolic liver diseases.

## 1. Introduction

Metabolic dysfunction-associated steatotic liver disease (MASLD) has become one of the most common chronic liver diseases worldwide, affecting 25% of the world’s population as a result of lifestyle changes such as increased consumption of energy-dense diets and sedentary habits [1]. Metabolic steatohepatitis (MASH) is a severe form of MASLD with a potentially progressive course that can lead to liver fibrosis, cirrhosis, and hepatocellular carcinoma (HCC) [2,3]. The progression of MASLD is difficult to visualize in the early stages of the disease because of uncertainties related to the accuracy of noninvasive markers of liver damage and the lack of effectiveness data related to treatment in patients with MASLD [4]. A growing number of drugs to treat patients with MASLD are being tested in clinical trials [5]. However, despite years of efforts by researchers around the world, only the thyroid hormone receptor β (THR-β) agonist resmetirom and GLP-1 inhibitor semaglutide, which were approved in 2025, have shown great efficiency in patients with MASLD; these were approved for the treatment of MASLD by the Food and Drug Administration (FDA) [6,7]. A major obstacle to the development of drugs for MASLD is the absence of a clear understanding of the mechanisms underlying this metabolic disease [8].

The existing studies in the literature have suggested that MASLD results from a multi-hit pathogenesis [9]. The core driving risk of MASLD progression can be attributed to the accumulation of free fatty acids (FFAs), which either enter the mitochondria to undergo β-oxidation or are esterified and subsequently stored as triglycerides (TG) [10,11]. Previous studies have shown that liver adaptation and mitochondrial flexibility are the main pathological processes in the early stages of MASLD development, which are subsequently lost in MASLD [12]. Thus, alterations in mitochondrial biogenesis and accumulation of damaged mitochondria may occur secondary to defects in the mitophagy pathway observed in liver tissues from patients with MASLD [4,13]. Mitochondrial swelling, cristae disruption, and impaired mitochondrial oxidative phosphorylation may be the markers of MASLD, and the hepatic mitochondrial mass and mitochondrial respiratory capacity are considered related to the transition from MASLD to MASH [14,15].

The function of the Rho-specific guanine nucleotide dissociation inhibitor (RhoGDI), encoded by the gene of *Arhgdia*, has been previously reported to be critically modulated by cholesterol-enriched membrane domains like lipid rafts, which serve as targeted platforms for GTPase delivery and facilitate localized release of active GTPases by integrating biophysical cues and extracellular signals such as integrin engagement [16,17]. Therefore, RhoGDI is ubiquitously expressed and interacts with several Rho GTPases. RhoGDI acts as a master regulator, orchestrating the spatiotemporal activity of Rho kinases. Rho-kinase has been suggested to ameliorate metabolic disorders through activation of the adenosine monophosphate (AMP)-activated protein kinase (AMPK) pathway, suggesting that Rho-kinase is a novel therapeutic target of metabolic disorders [18]. However, previous studies have not clarified whether the Rho GTPase companion RhoGDI plays a similar role in MASLD. Moreover, the mechanism underlying the effects of RhoGDI and its regulatory role in lipids and mitochondrial function are not clear.

In this study, we investigated the role of RhoGDI in metabolic regulation by generating liver-specific knockout of its encoding gene *Arhgdia* in mice. Our results demonstrate that hepatic RhoGDI deficiency attenuates lipid accumulation, inflammation, steatosis, and fibrosis in mice fed with MASLD-inducing diets. Furthermore, we revealed that knockdown of RhoGDI decreased liver lipid accumulation by enhancing mitochondrial function and promoting the phosphorylation of AMPK. In addition, we observed that a natural compound, TR08, which has been reported to be a drug candidate in antihypertension [19], attenuated hepatic lipid accumulation, inflammation, and hepatic steatosis and fibrosis in mice. Mechanistically, targeting RhoGDI with TR08 yielded protective effects by enhancing mitochondrial function and decreasing liver lipid accumulation and fibrosis, indicating a mechanism promoting the phosphorylation of AMPK and mitochondrial function. These data identified RhoGDI as a potential target of MASLD treatment and demonstrated that TR08 is a promising drug candidate that can ameliorate MASLD by promoting fatty acid oxidation and lipid clearance.

## 2. Results

### 2.1. Hepatocyte Arhgdia Knockout Attenuated MASLD Progression by Improving Lipid Metabolism and Reducing Fibrosis

**Figure 1:**
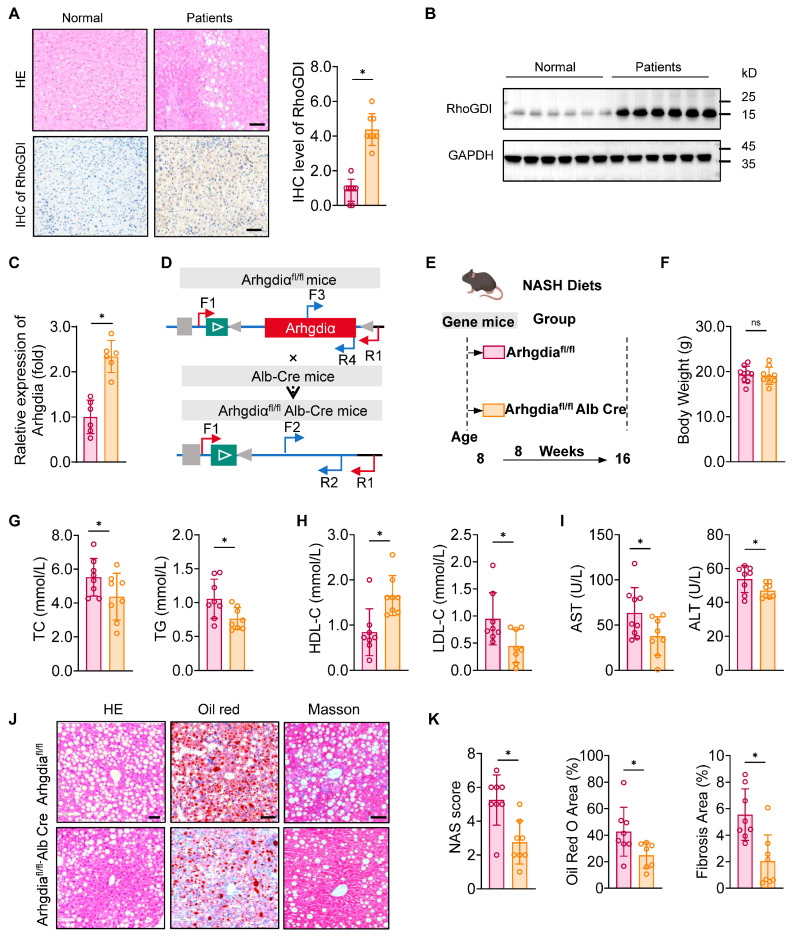
*Arhgdia* expression is upregulated in fatty liver during MASLD progression. (**A**) The protein expression level of RhoGDI is significantly elevated in the liver tissues of patients (n = 8); scale bar, 100 μm. (**B**) Western blot of RhoGDI in patient liver tissues. (**C**) Quantification of Arhgdia in mRNA level. (**D**) Schematic representation of the protocol for obtaining *Arhgdia^fl/fl^* Alb-Cre mice by the crossing Alb-Cre mice and *Arhgdia^fl/fl^* mice. (**E**) Schematic representation of experimental modeling of *Arhgdia^fl/fl^* Alb-Cre and *Arhgdia^fl/fl^* mice and group allocation with GAN diets. Quantification of body weight (**F**) and serum TG, TC (**G**), HDL-C, LDL-C (**H**), ALT, and AST levels (**I**). (**J**) Representative images of H&E staining (left), Oil Red O staining (middle), and Masson staining (right) of the liver tissues from *Arhgdia^f/f^* and *Arhgdia^f/f^* Alb-Cre mice. (**K**) Quantification of NAS, Oil Red O-stained area, and fibrosis area of liver tissues from *Arhgdia^f/f^* and *Arhgdia^f/f^* Alb-Cre mice. Scale bar, 50 μm (n = 8). Data are presented as mean ± SEM, * *p* < 0.05 according to Student’s *t* test.

To investigate the role of *Arhgdia* in fatty liver development, we first analyzed RhoGDI expression in human hepatic tissues with MASLD. Notably, RhoGDI exhibited much higher levels of expression in fatty livers than in normal tissues (Figure 1A), indicating a potential association with hepatic lipid metabolism. Furthermore, Both the mRNA and protein expression levels of RhoGDI were significantly upregulated in fatty livers than in normal tissues (Figure 1B,C). Hepatocyte-specific *Arhgdia*-knockout mice were obtained by crossing *Arhgdia^fl/fl^* mice with *Alb-Cre* transgenic mice, which were then fed high-fat and high-cholesterol diets to induce MASLD (Figure 1D,E). The mice with *Arhgdia* deletion did not show significant changes in body weight in MASLD (Figure 1F). However, *Arhgdia^fl/f,Alb-Cre^* mice showed marked improvements in serum lipid profiles, with reduced total cholesterol (TC) and TG levels (Figure 1G). Furthermore, these mice showed increased high-density lipoprotein cholesterol (HDL-C) levels and decreased low-density lipoprotein cholesterol (LDL-C) levels (Figure 1H). These findings suggest that *Arhgdia* deficiency mitigates hepatic lipid accumulation. Additionally, the levels of serum markers of liver injury, including aminotransferase (AST) and alanine transaminase (ALT), were significantly lower in *Arhgdia^fl/fl-Alb-Cre^* mice (Figure 1I). Histological analyses revealed reduced lipid deposition and fibrosis in *Arhgdia^fl/fl-Alb-Cre^* mice (Figure 1J,K). Collectively, our data highlight *Arhgdia* as a critical regulator of hepatic lipid metabolism and fibrosis, with its ablation conferring protection against MASLD progression.

### 2.2. Transcriptome Analysis Revealed That Arhgdia Ameliorates Hepatic Steatosis and Reduces Lipid Accumulation

**Figure 2:**
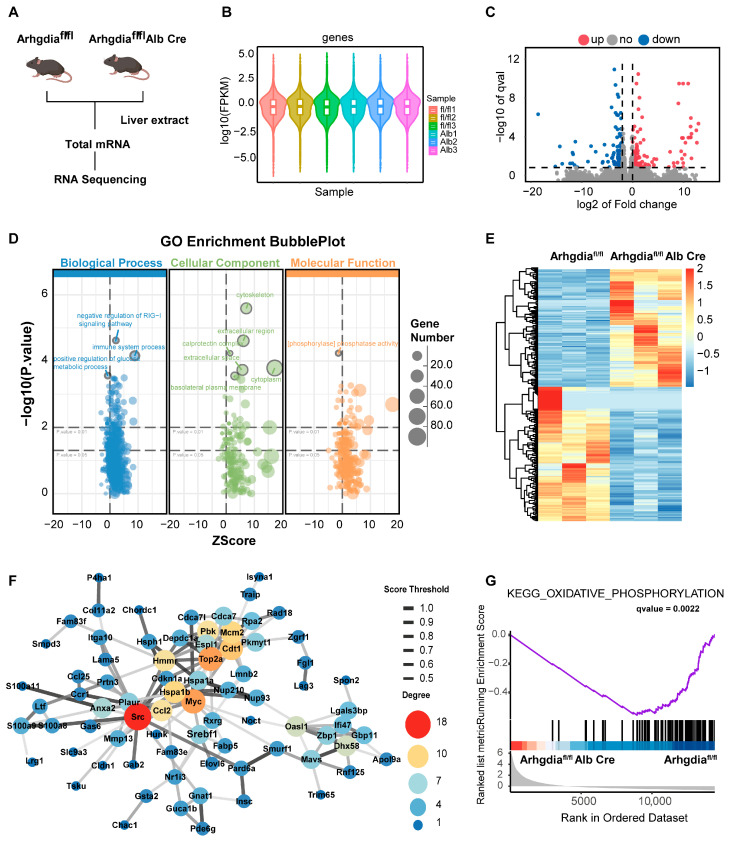
Hepatocyte-specific deletion of *Arhgdia* was shown to ameliorate hepatic steatosis and lipid deposition through mRNA deep sequencing. (**A**) Flow scheme of the mRNA deep sequencing from *Arhgdia^f/f^* and *Arhgdia^f/f^* Alb-Cre mice used for the MASLD model (n = 3). Violin plot of samples (**B**); a volcano plot (**C**). (**D**) Bubble chart of pathways by GO analysis. (**E**) Heatmap of the top 100 genes. (**F**) PPI network analysis of the top 100 genes. (**G**) GSEA enrichment of differential genes of the mouse model with *Arhgdia* deletion (n = 3). Data are presented as mean ± SEM.

To investigate the mechanistic role of *Arhgdia* in hepatic lipid metabolism, we conducted hepatocyte-specific RNA-seq analysis of liver tissues from *Arhgdia^fl/f,Alb-Cre^* mice and WT littermate controls (Figure 2A). Quality assessments revealed high reproducibility among biological replicates, as demonstrated by uniform distribution patterns in violin plots of gene expression profiles (Figure 2B). Differential expression analysis revealed 867 significantly upregulated genes and 521 downregulated genes in *Arhgdia*-deficient livers in comparison with WT controls (|log_2_FC| > 1, q Value < 0.05), and the results were visualized using a volcano plot (Figure 2C). Gene ontology (GO) enrichment analysis demonstrated significant enrichment of differentially expressed genes (DEGs) in biological processes related to glucose metabolism, extracellular matrix organization, and protein phosphorylation (Figure 2D). Hierarchical clustering of DEGs revealed distinct expression patterns between genotypes, which were visualized by heatmap analysis (Figure 2E). Protein–protein interaction (PPI) network analysis of these genes revealed key interacting proteins of the differential genes in the liver. Interestingly, *Srebf1* was shown to be related to lipid metabolism, especially cholesterol metabolism (Figure 2F). Moreover, GSEA further identified differential changes in *Arhgdia*-associated genes involved in mitochondrial oxidative phosphorylation pathway (Figure 2G). These findings collectively indicate that *Arhgdia* directly regulates genes involved in cholesterol metabolism and the mitochondrial oxidative phosphorylation in hepatocytes.

### 2.3. Arhgdia Knockdown Reduces Lipid Accumulation and Enhances Mitochondrial Function in Hepatocytes

**Figure 3:**
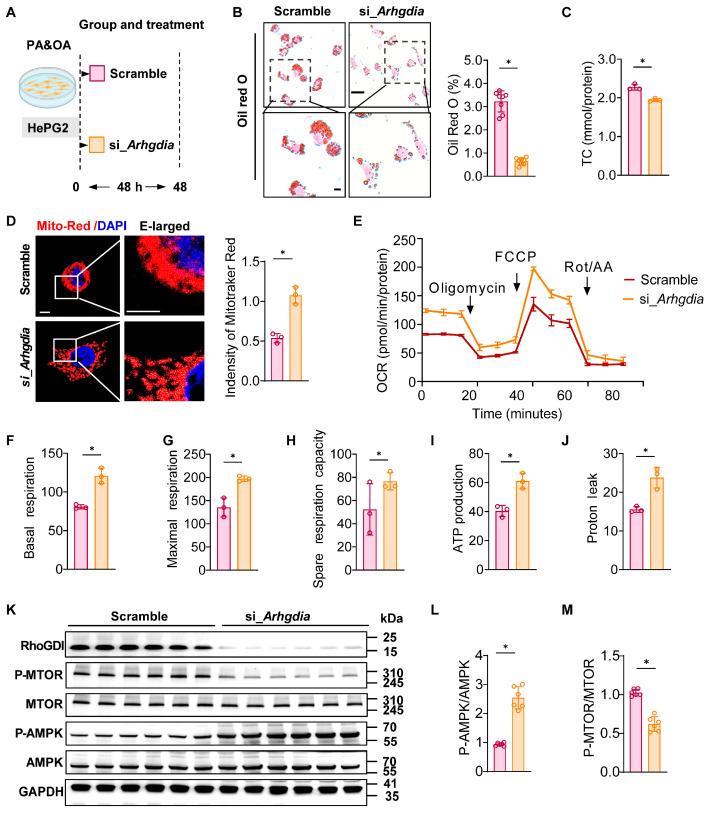
*Arhgdia* ameliorates hepatic steatosis through the mitochondrial AMPK/mTOR pathway. (**A**) Schematic diagram of in vitro experiments (Red indicates the scramble group; orange, the siArhgdia group). (**B**) Oil Red O staining of *Arhgdia* knockdown in PA/OA-induced models in HepG2 cells; scale bar: 10 μm. The lower position is the enlarged image of the upper position. (**C**) The TC level in the HepG2 cells transfected with *Arhgdia* siRNA. (**D**) Labeling mitochondria by Mito tracker Red; scale bar, 10 nm (n = 3). (**E**) Seahorse XFe96 analysis of cell respiration in HepG2 cells treated with TR08 followed by PA/OA or BSA administration for 24 h (n = 6). Basal respiration capacity of HepG2 cells (**F**). Maximal respiration capacity (**G**), spare respiration capacity (**H**), ATP production (**I**), and proton leakage (**J**) (n = 3). (**K**) Western blot analysis of total and phosphorylated protein levels of AMPK and mTOR in HepG2 cells transfected with si-*Arhgdia* and scrambled siRNA followed by treatment with PA/OA or the BSA vehicle for 24 h. (**L**,**M**) Western blot analysis of total and phosphorylated protein levels of p-AMPK/AMPK and p-mTOR/mTOR using ImageJ 15.0 software. Data are presented as mean ± SEM, * *p* < 0.05 by the *t* test.

To investigate the role of *Arhgdia* in lipid metabolism, we first established a PA/oleic acid (OA)-induced cellular model in HepG2 cells (Figure 3A). We found that *Arhgdia* knockdown significantly reduced lipid accumulation in hepatocytes under PA/OA-induced steatotic conditions (Figure 3B). Additionally, TC levels were markedly decreased in these hepatocytes (Figure 3C). Mito tracker red staining revealed an increased mitochondrial number in the cells treated with small interfering RNA (siRNA) targeting *Arhgdia* (Figure 3D). Given the critical role of mitochondria in lipid metabolism, we assessed the OCR using Seahorse XFe96 Analyzer. Cells transfected with *Arhgdia* siRNA exhibited stronger mitochondrial respiratory capacity, particularly in basal and maximal respiration (Figure 3E–G). Moreover, these cells also showed elevated spare respiratory capacity and ATP production (Figure 3H–J). Concurrently, we measured the phosphorylation levels of AMPK and mammalian target of rapamycin (mTOR). *Arhgdia* knockdown led to a significant increase in AMPK phosphorylation, while the levels of phosphorylated mTOR remained were markedly reduced (Figure 3K–M).

### 2.4. TR08 Ameliorates HFD-CCl4-Induced Metabolic Steatohepatitis in Mice

**Figure 4:**
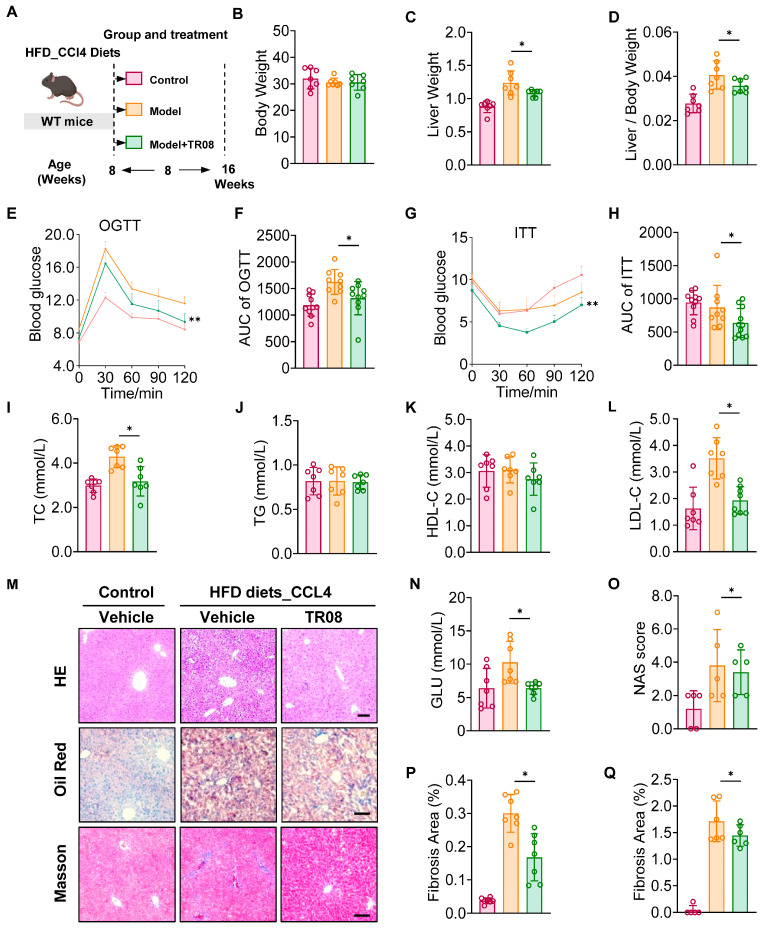
TR08 treatment ameliorates lipid accumulation and fibrosis in the HFD- and CCl_4_-induced mouse model. (**A**). Schematic diagram of the experimental procedure used to examine the protective effects of TR08 in mice fed an HFD diet for 12 weeks (The red represents the control group, the orange represents the model group, and the green represents the model plus TR08 group). Body weight (**B**), liver weight (**C**), and liver index (**D**) were evaluated in mice. The OGTT level (**E**) and the area under curve (AUC) of the OGTT (**F**) in mice were evaluated during the final week of HFD feeding. The ITT level (**G**) and area under the curve (AUC) of the ITT (**H**) in mice were evaluated during the final week of the HFD feeding. The TC level (**I**) and TG level (**J**), as well as HDL-C (**K**) and LDL-C levels (**L**), were measured in the serum of mice. n = 8. Data are presented as mean ± SEM, * *p* < 0.05, and ** *p* < 0.01 according to one-way ANOVA. (**M**) Representative images of H&E staining, Oil Red O staining, and Masson staining in liver sections from mice in the control, model, and TR08-treated groups. Scale bar, 50 μm. (**N**–**Q**). The glucose level and the evaluation of the staining results (**M**). n = 6. Data are presented as mean ± SEM, * *p* < 0.05, and ** *p* < 0.01 by one-way ANOVA.

To evaluate the therapeutic potential of the natural compounds in MASLD, we established a murine model by feeding male mice an HFD for 12 weeks, combined with intraperitoneal CCl_4_ injections followed by the last 4 weeks of HFD feeding, the control group was on a normal diet, while the administration group received TR08 via oral gavage under modeled conditions (Figure 4A). We found that TR08 (10 mg/kg/d) administration did not significantly alter body weight (Figure 4B). However, the mice showed significant reductions in liver weight (Figure 4C) and liver-to-body weight ratio (Figure 4D). Oral glucose tolerance tests (OGTTs) revealed improved glucose metabolism in TR08-treated mice after 16 weeks (Figure 4E,F). Meanwhile, the ITT curve for the condition with insulin injection and the area under the curve of the ITT curve showed an improvement in insulin sensitivity in response to TR08 treatment (Figure 4G,H). TR08 administration significantly reduced the TC and LDL-C levels in the serum of mice. No marked changes were observed in the TG or HDL-C levels of mice (Figure 4I–L). Hematoxylin and eosin (H&E) staining and Oil Red O staining of liver sections showed a reduction in lipid accumulation in the TR08-treated group in comparison with the model group. Masson staining showed a markedly reduced fibrosis area in liver tissues (Figure 4M). In parallel with the decrease in blood glucose levels post-administration (Figure 4N) in serum, the NAS (Figure 4N), the Oil Red O area (Figure 4P), and the fibrosis area (Figure 4Q) were simultaneously reduced. Collectively, these results demonstrated the therapeutic efficacy of TR08 in ameliorating MASLD progression in various mouse models.

**Figure 5:**
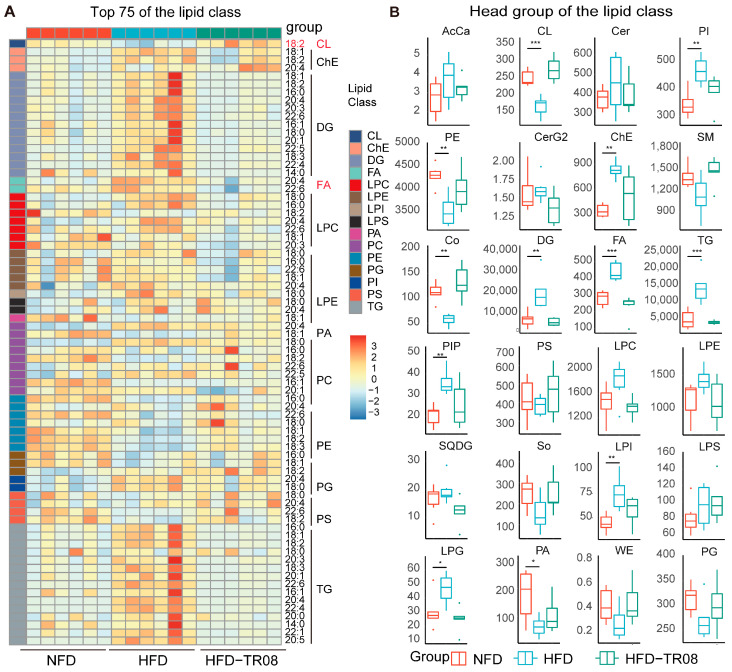
Pharmacological intervention with TR08 improves lipid accumulation in the liver. (**A**) Heatmaps of the lipidomic findings in the liver of the mice in the NFD group, HFD group and the group that received TR08 treatment (HFD-TR08 group). The colored boxes, ranging from blue to red, show the increasing abundance of lipids. (**B**) A column diagram of the head group of the lipid class with different groups (n = 6). The skewed variables are presented as the median with the interquartile range. * *p* < 0.05, ** *p* < 0.01 and *** *p* < 0.001 according to one-way ANOVA.

Lipidomics, based on high-throughput analytical technologies, enables systematic characterization of the composition and expression profiles of lipids in biological systems. To investigate the effects of TR08 on fatty acid metabolism, we performed untargeted lipidomic analysis of liver tissues. As shown in the heatmap in Figure 5A, lipids in the livers of TR08-treated mice were significantly downregulated compared with those in the control group, with a notable reduction in lipids containing fatty acid chains longer than 18 carbons, indicating impaired elongation of C18 and longer-chain fatty acids. In particular, significant downregulation of free fatty acids, triglycerides (TGs), and long-chain fatty acids was observed, while a marked upregulation of cardiolipin (CL) was also noted which reveals the mitochondrial relationships (Figure 5B). These findings suggest that TR08 treatment significantly downregulates hepatic lipid metabolism and exerts its ameliorative effects on MASLD by disrupting lipid metabolic homeostasis.

### 2.5. TR08 Attenuates MASLD Progression by Enhancing Lipid Disposion Through Arhgdia

**Figure 6:**
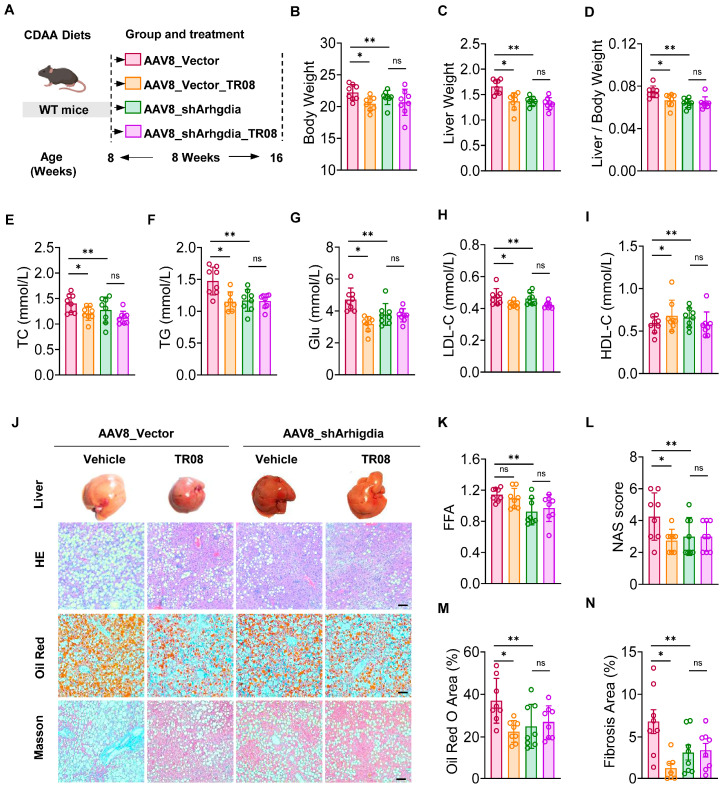
*Arhgdia* silencing attenuates hepatic steatosis in mice. (**A**) Schematic diagram of the experimental procedure used to examine the protective effects of TR08 in mice fed CDAA diets for 8 weeks. Male mice were fed a CDAA diet for 8 weeks and injected with AAV8 expressing *Arhgdia* shRNA (AAV8-sh-*Arhgdia*) or a negative control, shRNA (AAV8-vector), through the tail vein, followed by 8 weeks of CDAA feeding (Red designates the AAV8_Vector group, orange denotes the AAV8_TR08 group, green indicates the shArhgdia group, and purple represents the group receiving combined AAV8 and TR08 treatment.). (**B**) Body weight. Liver weight (**C**) and liver index (**D**). TC (**E**), TG (**F**), glucose (**G**), LDL-C (**H**), and HDL-C (**I**) levels in serum, detected and normalized to liver weight. (**J**) Representative images of H&E staining and Oil Red O staining of liver sections. Scale bar, 50 μm. (**K**) Liver FFA level of the treated mice. (**L**) The NAS. (**M**) Quantification of Oil Red O staining. (**N**) Quantification of fibrosis area. n = 8. Data are presented as the mean ± SEM, * *p* < 0.05, and ** *p* < 0.01 according one-way ANOVA.

To investigate whether TR08 attenuates MASLD progression through *Arhgdia*, mice were fed MASLD diets, followed by TR08 administration for an additional 8 weeks while they continued to receive MASLD diets (Figure 6A). This model was expected to show substantially higher hepatic steatosis and more inflammation. We observed that the reductions in body weight, liver weight, and the liver weight-to-body weight ratio of HFD-fed mice were significantly affected by infection with sh-*Arhgdia* compared to the AAV8_vector group. Administration of TR08 significantly reduced the body weight, liver weight, and liver weight-to-body weight ratio compared to those of the AAV8_vector group, but these changes were not observed when mice underwent both TR08 administration and *Arhgdia* transfection compared to the AAV8_sh-*Arhgdia* group (Figure 6B–D). Meanwhile, the TC, TG, and Glu levels in serum, as well as the LDL-C levels and HDL-C levels in serum, reduced after infection with sh-*Arhgdia* compared to those of the AAV8_vector group (Figure 6E–I). These changes showed similar trends in response to TR08 treatment, but these changes were not observed when mice underwent both TR08 administration and *Arhgdia* transfection compared to the AAV8_sh-*Arhgdia* group. In addition, H&E staining showed that hepatocyte ballooning was reduced in mice treated with TR08 in comparison with those that received the AAV8 vector. However, hepatocyte ballooning was similar in mice treated with TR08 and those injected with AAV8-sh-*Arhgdia* (Figure 6J). Additionally, the liver FFA level decreased in response to TR08 treatment in comparison with that in the AAV8-vector group. The *Arhgdia* and *Arhgdia* + TR08 groups underwent identical injections, with TR08 was added only later. However, the two groups did not show significant differences in liver FFA levels (Figure 6K). Pathological assessment, including the NAS, Oil Red O staining, and fibrosis analysis, consistently indicated that TR08 treatment ameliorated liver steatosis, inflammation, and injury compared to the AAV8-vector control group. Notably, the Arhgdia and Arhgdia + TR08 groups received identical viral injection, with TR08 administration commencing only afterward. Despite this treatment difference, the two groups did not exhibit statistically significant differences in pathological assessment (Figure 6L–N). These results suggest that TR08 regulates lipid metabolism and liver injury through RhoGDI to enhance mitochondria function and reduce lipid accumulation in the liver, thereby delaying the progression of MASLD.

### 2.6. TR08 Ameliorates Lipid Accumulation by Enhancing Mitochondria Function Through Arhgdia

**Figure 7:**
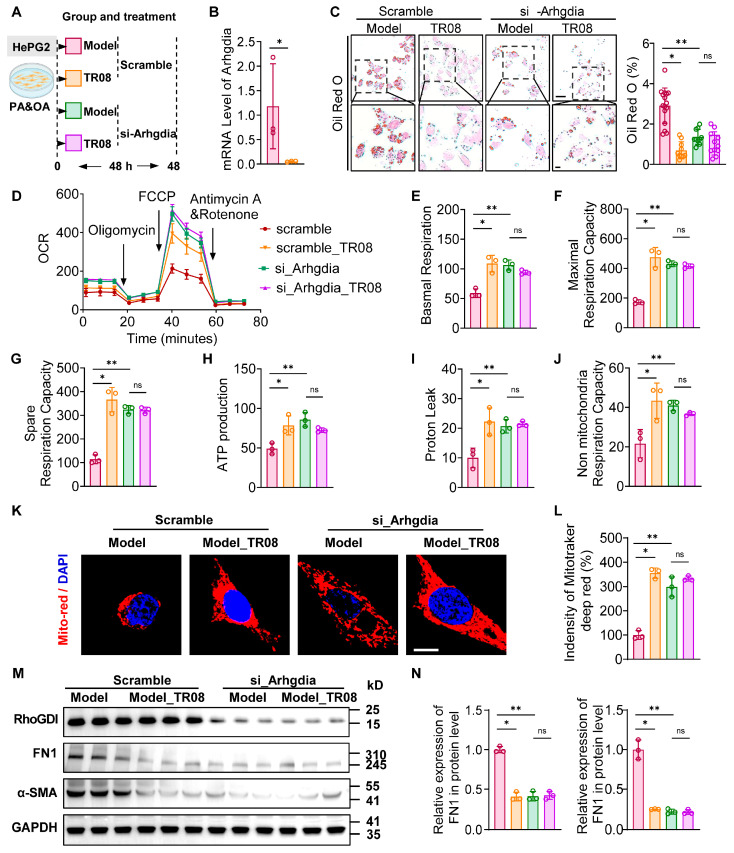
TR08 administration increases energy consumption and mitochondrial function in HepG2 cells. (**A**) The schematic flow chart of the in vitro experiment (The colors correspond to the following groups: red, model group; orange, TR08 treatment group; green, siArhgdia group; purple, siArhgdia with TR08 treatment group). (**B**) Oil Red O staining of HepG2 cells induced by PA/OA, administered TR08, and transfected with *Arhgdia* siRNA. (**C**) Statistical analysis of the positive area of Oil Red O staining (**B**). Scale bar, 10 μm. (**D**) Seahorse XFe96 analysis of respiration of HepG2 cells after induction by PA/OA with administration of TR08 and transfer of *Arhgdia* siRNA. (**E**) Basal respiration capacity of HepG2 cells. Maximal respiration capacity (**F**), spare respiration capacity (**G**), ATP production (**H**), proton leak (**I**), and non-mitochondria capacity of respiration (**J**). (**K**) Labeling of mitochondria by Mito tracker Red; scale bar, 10 nm. (**L**) Quantitative analysis of the fluorescence intensity of Mito tracker Red. (**M**) The mRNA level of *Arhgdia* in HepG2 cells infected with *si-Arhgdia*. (**N**) Western blot analysis of protein levels of FN1 and α-SMA in the cells that received TR08 followed by treatment with PA or the BSA vehicle for 24 h. n = 3. Data are presented as the mean ± SEM, * *p* < 0.05, and ** *p* < 0.01 according to one-way ANOVA.

To investigate the mechanism of TR08, we employed a lipid accumulation model in vitro via the administration of PA/OA in HepG2 cells (Figure 7A). The *Arhgdia* knockdown mice were observed and revealed a decrease in Arhgdia in HepG2 cells (Figure 7B). We found that lipid accumulation in HepG2 cells was significantly reduced by TR08 treatment. Notably, the extent of this reduction varied across *Arhgdia* knockdown conditions. Oil Red O staining of PA/OA-treated cells confirmed that TR08 intervention effectively decreased lipid deposition (Figure 7C). Given the central role of mitochondria in lipid metabolism, we measured the OCR using Seahorse XFe96 Analyzer. HepG2 cells transfected with *Arhgdia* siRNA exhibited enhanced mitochondrial respiratory capacity, particularly in basal and maximal respiration compared to the scramble group, while the TR08 treatment reduced mitochondrial function in response to *Arhgdia* siRNA (Figure 7D–G). Additionally, spare respiratory capacity and ATP production revealed TR08 enhanced mitochondria function (Figure 7H–J). These findings indicate that si-*Arhgdia* enhanced mitochondrial function, while TR08 administration caused a marked increase in mitochondrial function, suggesting an increase in oxidative phosphorylation during FFA metabolism. Subsequent mitochondrial functional assays demonstrated that *Arhgdia* knockdown significantly improved mitochondrial activity (Figure 7K). Although TR08 alone also enhanced mitochondrial function, its effects on liver lipid levels were lower than those observed in response to *Arhgdia* knockdown. In fact, early intervention targeting mitochondria showed potential in preventing or slowing fibrosis progression. We also evaluated fibrosis markers and found that *Arhgdia* knockdown significantly reduced the levels of fibronectin 1 (FN1) and α-smooth muscle actin (SMA) (Figure 7M,N). These results indicate that *Arhgdia* inhibits liver fibrosis by enhancing the mitochondrial function of hepatocytes.

## 3. Discussion

In this study, we found that RhoGDI is upregulated in the liver of patients and that hepatocyte-specific knockout of *Arhgdia* improves liver lipid accumulation and fibrosis in mice by enhancing AMPK phosphorylation and mitochondrial function and reducing lipid accumulation. Additionally, we identified a natural compound, TR08, that targeted RhoGDI to protect against the progression of MASLD in mice. Subsequently, infection with *Arhgdia* shRNA AAV8 ameliorated lipid accumulation and fibrosis in the liver. Meanwhile, TR08 also ameliorated lipid accumulation and fibrosis, providing further evidence for the efficacy of targeting RhoGDI with TR08. We further demonstrated that TR08 improves mitochondrial function through RhoGDI by administering TR08 to cells and knocking down RhoGDI.

RhoA has been shown to mitigate hepatic lipid accumulation in MASLD, yet its broader pathophysiological roles in inflammation and fibrosis remain unclear [20]. Further investigation into its natural inhibitor, RhoGDI, which is implicated in fibrosis and cardiovascular disease, is necessary [21]. Investigating RhoGDI could reveal deeper mechanistic insights into RhoA’s function in metabolic liver disease. In this study, we found that RhoGDI also plays an important role in preventing MASLD progression. RhoGDI improved liver lipid accumulation and fibrosis in mice by enhancing AMPK phosphorylation and mitochondrial function and reducing lipid accumulation. The compound, TR08, interacted with RhoGDI to significantly alleviate hepatic steatosis, fibrosis, and inflammation in the mouse model used. Mechanistically, TR08 not only decreased hepatic steatosis by enhancing lipid degradation, but also decreased fibrosis, thus preventing MASLD progression. Lipid accumulation is characteristic of liver steatosis, and evidence indicates that increased fatty acid uptake is associated with lipid accumulation [22]. In the present study, treatment with TR08 ameliorated hepatic lipid accumulation and fibrosis. Slowing down the tricarboxylic acid (TCA) cycle impairs fatty acid oxidation, resulting in fat accumulation in the liver. In this regard, elevated plasma levels of TGs and FFAs may contribute to HFD-induced body weight gain, while HFD-triggered glucose intolerance and insulin resistance play key roles in obesity and systemic metabolic dysfunction in mice.

Over the past decade, the discovery of AMPK substrates has improved the understanding of cellular metabolism from anabolism to catabolism, and AMPK has been identified as a central mediator of cellular metabolism that coordinates multiple features of metabolism as well as the mitochondrial response to energetic stress and mitochondrial insults [23]. The genetic and pharmacological disruption of RhoA activity has been shown to effectively mitigate MASLD phenotypes in mice [24]. Researchers have reported that the AMPK-independent pathway may be regulated by Rho GTPase, although the detailed mechanisms underlying this finding remain to be elucidated [25,26,27]. However, our findings for RhoGDI indicate the mechanism of regulation of AMPK in metabolism. We observed that knockdown of RhoGDI promoted the phosphorylation of AMPK, and that TR08 targeted RhoGDI by activating the AMPK pathway. In conclusion, our results highlighted a regulatory mechanism based on RhoGDI and identified a molecule that could facilitate metabolic regulation. TR08 showed protective effects against lipid deposition and fibrosis in a murine MASLD model. Moreover, TR08 activated the AMPK pathway, which decreased FFA synthesis and promoted mitochondrial activation in male mice by targeting *Arhgdia*. TR08 reduced progressive liver fibrosis, providing a potential therapeutic strategy for MASLD treatment. The RhoGDI-targeting approach and the mechanism underlying AMPK regulation can serve as the foundation for new drug design in MASLD treatment.

## 4. Materials and Methods

### 4.1. Animals

All mice were housed in isolated ventilated cages in an animal barrier facility at the Hefei University of Science and Technology (Hefei, China). The mice were maintained on a 12/12 h light/dark cycle at 22 °C ± 2 °C with ad libitum access to pellet food and water. Adult male mice were used at various ages, as indicated for the respective experiments.

### 4.2. Ethics Statements

Animal studies were conducted in compliance with the recommendations in the Guidelines on the Care and Use of Animals for Scientific Purposes of the National Advisory Committee for Laboratory Animal Research. All experimental procedures were approved by Hefei University of Technology (HFUT20210503001). A trained researcher was responsible for conducting all animal experimental procedures in accordance with the laws governing animal research in China. A total of 16 adults who underwent liver biopsy for suspected MASLD were also enrolled in this analysis. The study was approved by the Research Ethics Committee of Putuo Hospital, Shanghai University of Traditional Chinese Medicine (No. PTEC-A-2025-13(S)-1). The severity of liver steatosis, inflammation, and fibrosis was assessed using the non-alcoholic fatty liver disease (NAFLD) activity score (NAS) [28] and NAFLD fibrosis stage by an experienced senior pathologist who was blinded to the clinical information of the patients.

### 4.3. Generation of Genetically Modified Mice

*Arhgdia^fl/+^* mice were purchased from GemPharmatech Co., Ltd. (Shanghai, China). *Arhgdia^fl/fl^* mice were generated using the CRISPR/Cas9 system and Cre-loxP-mediated recombination technology. First, two single-guide RNAs (sgRNA1 and sgRNA2) were used to target a fragment of *Arhgdia* exon 1–6. Then, the single-stranded oligodeoxynucleotides (ssODNs), sgRNAs, and Cas9 mRNA were injected into zygotes (GenePharmatech, Shanghai, China). We selected one founder mouse and crossed it with a C57BL/6J mouse to generate *Arhgdia^fl/fl^* mice. *Arhgdia ^fl/fl^* mice were mated with B6.FVB (129)-Tg (Alb-cre) mice, where the Cre was directed to specifically express in the liver, to generate *Arhgdia ^fl/fl, Alb^* mice, and *Arhgdia ^fl/fl^* mice served as controls.

### 4.4. HFD Diets Feeding, AAV8 Injection, and Pharmacological Treatment

The MAFLD model was established by feeding 8-week-old C57/BL6N male mice at a high-fat diet (HFD; 60 kcal% fat, 20 kcal% protein, 20 kcal% carbohydrate; D12492; Research Diets, New Brunswick, NJ, USA) for 12 weeks in the evaluation of the efficiency of compound TR08 (10 mg/kg/d, og) in the model and the TR08 treated group. CCl_4_ was diluted in olive oil at a ratio of 1:9 to prepare a 10% (*v*/*v*) solution. Mice received injections at a dose of 5 µL/g body weight (equivalent to 0.5 µL/g of pure CCl_4_) twice per week for 4 weeks. A GAN diet (40 kcal% Fat, 20 kcal% Fructose and 2% Cholesterol; D09100310; Research Diets, New Brunswick, NJ, USA) was administered for 12 weeks in the *Arhgdia^fl/fl,Alb^* mice group, and *Arhgdia^fl/fl^* mice group. Mice received a tail vein injection of AAV8 (Vigene Biosciences, Jinan, China) at a dose of 1 × 10^12^ vg/mL. The treatment for all experimental groups consisted of either the AAV8 construct or a control plasmid. Mice that were fed with a normal control (NC) diet (Q031, Shanghai Xietong Biologic Science & Technology, Shanghai, China) served as controls. Blood was collected from the retro-orbital plexus under isoflurane anesthesia. The collected whole blood was centrifuged for subsequent biochemical analysis.

### 4.5. Cell Culture and Treatment

The HepG2 cell line was cultured in Dulbecco’s modified Eagle medium (DMEM) supplemented with 10% fetal bovine serum, 100 μg/mL streptomycin, and 100 U/mL penicillin at 37 °C in a humidified atmosphere containing 5% CO_2_. To establish a cellular model of lipid accumulation, a modeling agent was prepared by complexing palmitic acid (PA) and oleic acid (OA) with 5% bovine serum albumin (BSA) to a final concentration of 5 mM for each fatty acid. This mixture was then applied to the culture system at a final concentration of 1 μM. Cells were subsequently treated with TR08 at a final concentration of 20 μM for 24 h. For in vitro coculture assays, HepG2 cells (obtained from Percell, Wuhan, China) were used. To investigate the function of Arhgdia, HepG2 cells at 60–70% confluence were transfected with either si-Arhgdia or a scramble control using Lipofectamine 3000 (L3000015, Thermo Scientific, Waltham, MA, USA) in serum-free medium according to the manufacturer’s protocol. After 6 h of transfection, the medium was replaced with complete medium to minimize cytotoxicity. Protein expression was evaluated 48 h post-transfection by Western blotting.

### 4.6. Western Blot Analysis

HepG2 cells were seeded in a 6-well plate and liver lysates were subjected to sodium dodecyl sulfate (SDS)-polyacrylamide gel electrophoresis, transferred to nitrocellulose or polyvinylidene difluoride (PVDF) membranes, and then blotted with antibodies. The immunoblots were visualized by chemiluminescence using an enhanced chemiluminescence Western blotting system (Bio-Rad Laboratories, Shanghai, China). The antibodies used in this study are indicated in Table 1. Quantification was performed by measuring the band intensities using Image J software version 2.14.0.

### 4.7. Glucose and Insulin Tolerance Tests

The oral glucose tolerance test (OGTT) was conducted one week before euthanasia. After a 16 h fast, the animals received a glucose solution (2 g/kg body weight) by oral gavage. For the insulin tolerance test (ITT), the animals were fasted for 6 h and then administered insulin (0.75 U/kg body weight) via intraperitoneal injection. Venous tail blood samples were collected at 0, 30, 60, 90, and 120 min post-administration, and blood glucose levels were measured using a glucose meter (OneTouch Verio, Johnson & Johnson, New Brunswick, NJ, USA).

### 4.8. Biochemical Analysis

The serum levels of aspartate AST, ALT, total TG, and total TC and Glucoses (GLU) were determined with an automated biochemical analyzer (Hitachi 7600, Tokyo, Japan), while TG (A110-1-1, Nanjing Jiancheng Bioengineering Institute, Nanjing, China) levels, TC (A111-1-1, Nanjing Jiancheng Bioengineering Institute, Nanjing, China) and hepatic FFA (A042-2-1, Nanjing Jiancheng Bioengineering Institute, Nanjing, China) levels were measured using commercial assay kits in accordance with the manufacturer’s instructions.

### 4.9. Immunohistochemistry Analysis

RhoGDI protein expression was assessed in liver tissues from eight patients and normal controls using Immunohistochemistry. Paraffin-embedded sections were subjected to antigen retrieval and incubated overnight at 4 °C with a primary antibody against RhoGDI (1:200, #EPR3773, Abcam, Waltham, MA, USA). Following incubation with a horseradish peroxidase-conjugated secondary antibody at room temperature for 1 h, immunoreactivity was visualized with 3,3′-diaminobenzidine substrate and counterstained with hematoxylin. Stained sections were examined and analyzed under a light microscope.

### 4.10. H&E Staining

For histological evaluation, liver tissue samples were fixed in 4% paraformaldehyde at room temperature for 24 h, dehydrated through a graded ethanol series, cleared in xylene, and embedded in paraffin. Consecutive sections of 5 μm thickness were cut using a microtome (Leica RM2235, Leica, Wetzlar, Germany). After deparaffinization and rehydration, the sections were sequentially stained with hematoxylin (D006-1-1, Nanjing Jiancheng Bioengineering Institute, Nanjing, China) for 5 min and eosin for 1 min. Following dehydration through an ethanol gradient and clearing in xylene, the sections were mounted with neutral resin. Stained sections were examined for histomorphology and images were captured under a light microscope (DM2500, Leica, Wetzlar, Germany).

### 4.11. Masson’s Trichrome Staining

Masson’s trichrome staining was performed to assess collagen deposition in liver tissues. Deparaffinized sections were mordanted in Bouin’s solution overnight, followed by sequential staining with Weigert’s iron hematoxylin (10 min), Biebrich scarlet-acid fuchsin (10 min), phosphomolybdic–phosphotungstic acid (10 min), and aniline blue (5 min). After dehydration and mounting, collagen fibers (blue), muscle fibers (red), and nuclei (blue-black) were visualized under a light microscope (DM2500, Leica, Wetzlar, Germany). The collagen area percentage was quantified using ImageJ software (version 1.53) from three randomly selected fields per section.

### 4.12. Oil Red O Staining

Liver cryosections (8–10 μm thick) were air-dried and fixed in pre-chilled 4% paraformaldehyde for 15 min, followed by a brief rinse in 60% isopropanol. Sections were then stained with a filtered Oil Red O working solution for 30 min at room temperature. After thorough rinsing in 60% isopropanol and distilled water to remove background dye, nuclei were counterstained with Mayer’s hematoxylin for 1 min. Finally, sections were washed under running tap water, and examined under a light microscope.

### 4.13. Mitochondrial Staining

HepG2 cells cultured on coverslips were incubated with MitoTracker™ Deep Red (M22672, Thermo Fisher Scientific, Waltham, MA, USA) at a working concentration of 100 nM in serum-free medium for 30 min at 37 °C in the dark. After staining, cells were washed twice with warm PBS, fixed with 4% paraformaldehyde for 15 min, and mounted with ProLong™ Gold Antifade Mountant containing DAPI. Fluorescence images were captured using a confocal microscope (Leica TCS SP8). Mitochondrial morphology and distribution were analyzed with ImageJ software.

### 4.14. Quantitative Real-Time PCR

Total RNA was extracted from tissue or cell samples using FreeZol Reagent (R711, Vazyme, Nanjing, China) following the manufacturer’s instructions. RNA was reverse-transcribed into cDNA using HiScript II Q RT SuperMix for qPCR (+gDNA wiper) (R223, Vazyme). Quantitative Real-Time PCR (qPCR) was performed with ChamQ SYBR qPCR Master Mix (Low ROX Premixed) (Q331, Vazyme) on a QuantStudio 5 Real-Time PCR System (Applied Biosystems, Waltham, MA, USA). The primer sequences were as follows: forward: CGGCCAAGAGGAAGGAGTAT; reverse: GCATCCTTGTCATTGGCTTT. Relative gene expression was normalized to GAPDH and calculated using the 2^−ΔΔCT^ method.

### 4.15. Lipidomic Analysis

Liver tissues (20 mg) were extracted with chloroform–methanol–water (2:1:0.8, *v*/*v*/*v*), dried under nitrogen, and reconstituted in isopropanol–acetonitrile (1:1). Separation was performed on an Agilent 1290 Infinity II UHPLC system using a ZORBAX RRHD Eclipse Plus C18 column (Agilent, Santa Clara, CA, USA) (1.8 μm, 2.1 × 100 mm, 45 °C) with a gradient of mobile phases A (acetonitrile-water with 10 mM ammonium formate) and B (isopropanol-acetonitrile with 10 mM ammonium formate) at 0.3 mL/min over 20 min. Detection was carried out on Agilent 6545 Q-TOF (Agilent, Santa Clara, CA, USA) in positive/negative switching mode (resolution ≥ 40,000, *m*/*z* 100–1700). Lipids were identified with LipidSearch 4.2 and quantified by normalization to internal standards (SPLASH^®^ LIPIDOMIX) and tissue weight. This experiment was completed/tested by Bioprofile (Shanghai, China).

### 4.16. Transcriptomic Analyses

The Transcriptomic analyses were performed by LC-Bio Technology Co., Ltd. (Shanghai China). Using Trizol reagent, total RNA was isolated from the liver tissues of *Arhgdia^fl/fl,Alb^* mice and *Arhgdia ^fl/fl^* mice with NASH diets, as shown in Figure 1. The RNA quality was checked with Bioanalyzer 2200 (Agilent Technologies, Santa Clara, CA, USA). cDNA libraries were prepared using NEBNext^®^ Ultra™ Directional RNA Library Prep Kit, NEB Next^®^ Poly (A) mRNA Magnetic Isolation Module, and NEB Next^®^ Multiplex Oligos according to the instructions of the manufacturer (New England Biolabs, Ipswich, MA, USA). Genes with fold-change > 2.0 or <0.5 and a false discovery rate < 0.05 were considered to be significantly differentially expressed. Volcano, heat, and bubble maps were generated using the “ggplot2” packages (version 3.3.0) in R (version 3.6.3; The R Project for Statistical Computing, Vienna, Austria). Gene set enrichment analysis (GSEA) was performed using the “enrichplot” package (version 1.8.1).

### 4.17. Mitochondrial Respiration Analysis

HepG2 cells were plated in an XF96 plate (W21715; Seahorse Biosciences, North Billerica, MA, USA) at a density of approximately 1.0 × 10^4^ cells/well. The cells were equilibrated in DMEM containing 10 mmol/L glucose and 1 mmol/L sodium pyruvate. Hepatic mitochondrial respiration was measured by Seahorse Bioscience XFe 96 Extracellular Flux Analyzer. The baseline oxygen consumption rate (OCR) was recorded, which was followed by continuous injections of the following pharmacologic inhibitors through ports in the XF Assay cartridges: oligomycin (1 mM), an inhibitor of adenosine triphosphate (ATP) synthase that enabled measurements of ATP-coupled oxygen consumption through oxidative phosphorylation (OXPHOS); carbonyl cyanide 4-trifluoromethoxy phenylhydrazone (FCCP) (1.5 μM), an uncoupling agent that produced maximum electron transport and therefore enabled measurement of maximum OXPHOS respiration capacity; and a mixture of antimycin A (1 mM) and rotenone (1 mM), which are mitochondrial complex I and III inhibitors, respectively. The injections were performed during continuous oxygen measurements using Seahorse XFe96 Extracellular Flux Analyzer (Seahorse Biosciences, USA) according to the manufacturer’s instructions.

### 4.18. Statistical Analysis

Statistical analyses were performed using GraphPad Prism software (version 10.1.2). Continuous parameters were compared using one-way analysis of variance (ANOVA), except for the non-normally distributed data, which were compared using a Kruskal–Wallis H test. Categorical variables were compared using the chi-square test. For the animal and in vitro studies, the data were presented as the mean ± standard error of the mean. An unpaired, two-tailed Student *t* test was used for two-group comparisons, and one-way ANOVA was used for comparisons among multiple groups, which was followed by a least significant difference (LSD) post hoc test for the differences between any two groups. The asterisks in the figures indicate statistical significance as follows: * *p* < 0.05; ** *p* < 0.01.

## 5. Conclusions

This study demonstrate that hepatocyte-specific Arhgdia deletion confers protection against the progression of MASLD by reducing hepatic lipid accumulation and attenuating fibrotic development. Additionally, these results indicated that RhoGDI inhibition enhances mitochondrial β-oxidation in hepatocytes. These results establish RhoGDI as a critical regulator of MASLD pathogenesis and highlight its potential as a therapeutic target for metabolic liver diseases.

## Figures and Tables

**Table 1:** Antibodies used in this article.

Name	Description	Cat	Species	Company	Dilution
FN1	Fibronectin Rabbit mAb	A23830	Rabbits	ABclonal (Woburn, MA, USA)	1:2000
P-MTOR	Phospho-mTOR-S2448 Rabbit mAb	AP1413	Rabbits	ABclonal	1:1000
MTOR	mTOR Rabbit pAb	A24743	Rabbits	ABclonal	1:1000
P-AMPK	Phospho-AMPKα (Thr172) Antibody	2531S	Rabbits	Cell Signaling Technology (CST) (Danvers, MA, USA)	1:1000
AMPK	AMPK Alpha Polyclonal antibody	10929-2-AP	Rabbits	Proteintech (Rosemont, IL, USA)	1:40,000
GAPDH	GAPDH Monoclonal antibody	60004-1-Ig	Mouse	Proteintech	1:50,000
α-SMA	Alpha smooth muscle actin specific Monoclonal antibody	67735-1-Ig	Mouse	Proteintech	1:40,000
RHOGDI	RhoGDI antibody	EPR3773	Rabbits	ABCAM (Waltham, MA, USA)	1:2000

## Data Availability

The raw data supporting the conclusions of this article will be made available by the authors on the request.

## Abbreviations

The following abbreviations are used in this manuscript:

ALT	Alanine aminotransferase;
AMP	Adenosine monophosphate
AMPK	Adenosine monophosphate (AMP)-activated protein kinase
ANOVA	Analysis of variance;
AST	Aspartate aminotransferase;
ATP	Adenosine triphosphate;
BSA	Bovine serum albumin;
CL	Cardiolipin
DEG	Differentially expressed genes;
DMEM	Dulbecco’s modified eagle medium;
FCCP	Carbonyl cyanide 4-trifluoromethoxy phenylhydrazone
FFA	Free fatty acids;
GO	Gene ontology;
GSEA	Gene set enrichment analysis;
GTT	Glucose tolerance test;
H&E	Hematoxylin and eosin;
HCC	Hepatocellular carcinoma
HDL-C	High-density lipoprotein cholesterol
HFD	High-fat diet;
ITT	Insulin tolerance test;
LDL-C	Low-density-lipoprotein cholesterol
LSD	Least significant difference;
LW/BW	Liver weight-to-body weight ratio
MASH	Metabolic dysfunction-associated steatohepatitis;
MASLD	Metabolic dysfunction-associated steatotic liver disease;
mTOR	Mammalian target of rapamycin
NAFLD	Non-alcoholic fatty liver disease;
NAS	Non-alcoholic fatty liver disease activity score
NC	Normal control;
OCR	Oxygen consumption rate;
OGTT	Oral glucose tolerance tests;
OXPHOS	Oxidative phosphorylation;
PA	Palmitic acid;
PPI	Protein interaction;
PVDF	Polyvinylidene difluoride
qPCR	Quantitative Real-Time PCR
RhoGDI	Rho GDP-dissociation inhibitor;
SDS	Sodium dodecyl sulfate
siRNA	Small interfering RNA
SMA	Smooth muscle actin;
ssODNs	Single-stranded oligodeoxynucleotides
TC	Total cholesterol;
TCA	Tricarboxylic acid;
TG	Total triglyceride;
THR-β	Thyroid hormone receptor β
WT	Wild-type;
